# Supplementary material for: Towards an inclusive nature conservation initiative: Preliminary assessment of stakeholders’ representations about the Makay region, Madagascar
Source: PLoS One. 2022 Aug 26;17(8):e0272223. doi: 10.1371/journal.pone.0272223 (PMC9417016; doi:10.1371/journal.pone.0272223)
Supplement: S1 Table — (DOCX) [file pone.0272223.s002.docx]

S1 Table: List of the 10 references indexed in Scopus and dealing with the Makay region in Madagascar. References were identified by a search string [TITLE-ABS-KEY(Makay)] submitted on October 13, 2021 and by a screening of results’ titles and abstracts that allowed to exclude non-relevant references.

| **Identified references (with DOI when available)** |
| --- |
| Allorge L, Phillipson PB, Razakamalala R (2015) Catharanthus makayensis L. Allorge, Phillipson & Razakamal. (Apocynaceae), a new species from Madagascar. Candollea 70:61–66. <https://doi.org/10.15553/c2015v701a7> |
| Cargill DC, Callaghan DA, Forrest LL, Reeb C (2020) Fossombronia isaloensis Cargill & D.A.Callaghan, a new liverwort from sandstone massifs in southern Madagascar. J Bryol 42:213–222. <https://doi.org/10.1080/03736687.2020.1792672> |
| Csosz S, Loss AC, Fisher BL (2021) Taxonomic revision of the Malagasy Aphaenogaster swammerdami group (Hymenoptera: Formicidae). PeerJ 9:. <https://doi.org/10.7717/peerj.10900> |
| Gnezdilov VM (2021) New species of the genera Limentinus Distant, 1917 and Calodia Nielson, 1982 (Hemiptera, Auchenorrhyncha, Cicadellidae, Coelidiinae) from the Makay Massif of Madagascar, with a key to Malagasy species. Zoosystema 43:297–310. <https://doi.org/10.5252/zoosystema2021v43a16> |
| Gnezdilov VM (2020) A peculiar new species of the genus Grammacephalus Haupt (Hemiptera: Auchenorrhyncha: Cicadellidae: Deltocephalinae) from Madagascar. Zootaxa 4779:595–600. <https://doi.org/10.11646/zootaxa.4779.4.10> |
| Langer M, Boniface M, Cuny G, Barbieri L (2000) The phylogenetic position of Isalorhynchus genovefae, a Late Triassic rhynchosaur from Madagascar. Ann Paleontol 86:101–127. <https://doi.org/10.1016/S0753-3969(00)80002-6> |
| Laudisoit A, Prié V, Beaucournu J-C (2012) A new species o/Lagaropsylla Jordan & Rothschild, 1921 from Madagascar (Insecta, Siphonaptera, Ischnopsyllidae). Zoosystema 34:737–744. <https://doi.org/10.5252/z2012n4a5> |
| Manuel M, Ramahandrison AT (2020) Four new species of the diving beetle genus Laccophilus Leach, 1815 from Madagascar (Coleoptera, Dytiscidae, Laccophilini). Zootaxa 4822:485–502. <https://doi.org/10.11646/zootaxa.4822.4.2> |
| Rakotondravony HA, Goodman SM (2011) Rapid herpetofaunal surveys within five isolated forests on sedimentary rock in western Madagascar. Herpetol Conserv Biol 6:297–311 |
| Wesen T (2020) Ecotone shifts in southern madagascar: First barcoding data and six new species of the endemic millipede genus Riotintobolus (spirobolida, pachybolidae). ZooKeys 2020:1–29. <https://doi.org/10.3897/zookeys.953.53977> |
