# Supplementary material for: Towards an inclusive nature conservation initiative: Preliminary assessment of stakeholders’ representations about the Makay region, Madagascar
Source: PLoS One. 2022 Aug 26;17(8):e0272223. doi: 10.1371/journal.pone.0272223 (PMC9417016; doi:10.1371/journal.pone.0272223)
Supplement: S2 Table — (DOCX) [file pone.0272223.s003.docx]

S4 Table: Number of components and mean frequency for each component’s types, in the four zones of the Makay’s social representations

|  | **Number of components** | | **Mean frequency** |
| --- | --- | --- | --- |
| Central core zone | | | |
| Social components and processes | | 10 | 0,59 |
| New stakeholders | | 5 | 0,54 |
| Local people | | 2 | 0,78 |
| Agriculture and husbandry | | 1 | 0,88 |
| Incomes for local people | | 1 | 0,47 |
| Insecurity | | 1 | 0,28 |
| Biophysical components and processes | | 9 | 0,50 |
| Geomorphological features | | 3 | 0,55 |
| Species | | 2 | 0,48 |
| Makay's landscapes and habitats | | 2 | 0,47 |
| Access difficulties | | 1 | 0,63 |
| Erosion processes | | 1 | 0,28 |
| Ecosystem services | | 8 | 0,56 |
| Cultural ecosystem services | | 5 | 0,53 |
| Provisioning ecosystem services | | 3 | 0,60 |
| Negative human interventions | | 2 | 0,69 |
| Fires | | 1 | 0,97 |
| Environmental degradations | | 1 | 0,41 |
| Positive human interventions | | 2 | 0,44 |
| Conservation and development actions | | 2 | 0,44 |
| Contrasting elements’ zone | |  |  |
| Biophysical components and processes | | 7 | 0,20 |
| Geomorphological features | | 3 | 0,19 |
| Makay's landscapes and habitats | | 2 | 0,23 |
| Species | | 2 | 0,17 |
| Social components and processes | | 6 | 0,15 |
| Insecurity | | 1 | 0,19 |
| Food security issues | | 1 | 0,16 |
| New stakeholders | | 1 | 0,16 |
| Movements within Makay | | 1 | 0,13 |
| Social conflicts | | 1 | 0,13 |
| Sociopolitical processes | | 1 | 0,13 |
| Ecosystem services | | 3 | 0,16 |
| Provisioning ecosystem services | | 2 | 0,22 |
| Cultural ecosystem services | | 1 | 0,13 |
| Negative human interventions | | 2 | 0,69 |
| Environmental degradations | | 2 | 0,16 |
| Positive human interventions | | 2 | 0,44 |
| Conservation and development actions | | 2 | 0,14 |
| First periphery | |  |  |
| Social components and processes | | 8 | 0,56 |
| Agriculture and husbandry | | 3 | 0,66 |
| Cultural features | | 2 | 0,52 |
| New stakeholders | | 2 | 0,48 |
| Local people | | 1 | 0,53 |
| Biophysical components and processes | | 6 | 0,33 |
| Geomorphological features | | 3 | 0,34 |
| Species | | 2 | 0,31 |
| Surrounding landscapes | | 1 | 0,31 |
| Ecosystem services | | 5 | 0,39 |
| Provisioning ecosystem services | | 3 | 0,36 |
| Cultural ecosystem services | | 1 | 0,59 |
| Regulating ecosystem services | | 1 | 0,28 |
| Peripheral zone | |  |  |
| Biophysical components and processes | | 12 | 0,16 |
| Species | | 3 | 0,17 |
| Makay’s landscapes and habitats | | 3 | 0,15 |
| Erosion processes | | 2 | 0,20 |
| Climate | | 2 | 0,16 |
| Geomorphological features | | 2 | 0,13 |
| Social components and processes | | 11 | 0,17 |
| Authorities | | 3 | 0,19 |
| Demographic processes | | 2 | 0,17 |
| Sociopolitical processes | | 2 | 0.16 |
| Law enforcement | | 1 | 0,19 |
| Local people | | 1 | 0,19 |
| Media | | 1 | 0,19 |
| Agriculture and husbandry | | 1 | 0,13 |
| Ecosystem services | | 3 | 0,17 |
| Regulating ecosystem services | | 2 | 0,16 |
| Provisioning ecosystem services | | 1 | 0,19 |
| Negative human interventions | | 2 | 0,14 |
| Resources' external extraction | | 2 | 0,14 |
| Positive human interventions | | 1 | 0,13 |
| Conservation and development actions | | 1 | 0,13 |
| Ecosystem disservices | | 1 | 0,25 |
| Ecosystem disservices | | 1 | 0,25 |
