# Supplementary material for: Towards an inclusive nature conservation initiative: Preliminary assessment of stakeholders’ representations about the Makay region, Madagascar
Source: PLoS One. 2022 Aug 26;17(8):e0272223. doi: 10.1371/journal.pone.0272223 (PMC9417016; doi:10.1371/journal.pone.0272223)
Supplement: S1 Appendix — (DOCX) [file pone.0272223.s004.docx]

**S1 Appendix: Methods**

**Size of the sample of respondents**

In order to decide when to stop interviewing more respondents, we applied a data saturation technique that is commonly used in social investigations [1]: we considered that enough respondents were interviewed when no new information was added by an interview. More concretely in this study, we used the components cited by respondents to describe the Makay social-ecological system to determine saturation (S1 Fig.).

**
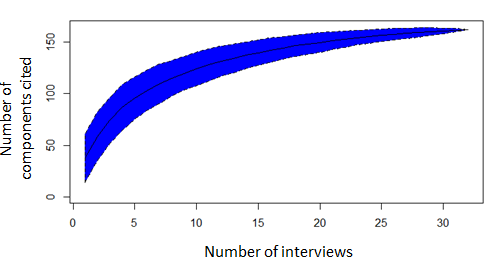
**

S1 Figure: Data saturation after 32 interviews.

**Direct elicitation procedure**

The direct elicitation procedure used in this study and describe in the article led to individual cognitive maps produced with the MentalModeler online software (<https://www.mentalmodeler.com>), exemplified in S2 Fig. below.

**
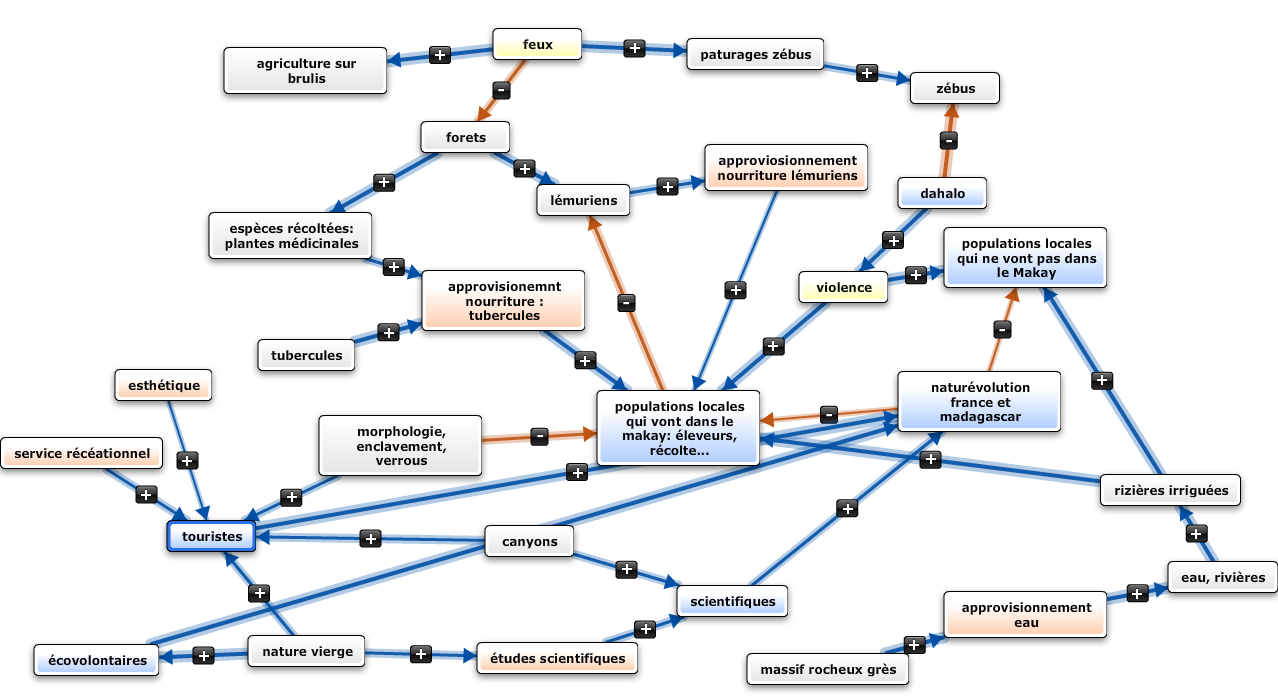
**

Dynamics, processes

and drivers of change

Actors

Ecosystem services

Characteristics


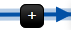

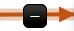


Positive interactions

Negative interactions

S2 Figure: Example of an individual cognitive map (in French) obtained at the end of an interview with the online software Mental Modeler [2].

**Standardized elicitation of mental models: semi-structured interview procedure**

*Preliminary information about the elicitation procedure*

The standardized elicitation procedure we developed was mainly inspired by the ARDI method that successively ask respondents to cite the Actors, Resources, Dynamics, and Interactions to describe their representation of an SES [3]. We selected this elicitation procedure for its proximity with word association techniques that are commonly used in social representation (SR) research to identify the content and structure of an SR [4,5]. Indeed, ARDI can be seen as a four-step word association technique, successively investigating respondents’ representations of different aspects of an SES (its actors, resources, dynamics and interactions). Yet, we modified the canvas of this elicitation procedure for three key reasons. First, instead of using ‘Resources’ which often refer to material features, we focused on ‘Ecosystem services’ as well as ‘Ecosystem disservices’ that encompass material and non-material features and that are more explicit than the resource concept regarding regulating ecological processes [6,7]. Second, and as a consequence of using the ecosystem service concept, we created a ‘Biophysical component and processes’ category which are the potential ecosystem service providers and which encompassed the Makay’s characteristics cited by respondents during the interview. Finally, we asked respondents to weigh the different interactions they cited as positive (+1) or negative (-1), contrary to the ARDI method that does not quantitatively weigh these interactions. This weigh was useful to quantitatively analyze individual cognitive maps and aggregate them into a social cognitive map in taking into account the degree of consensus among respondents regarding the positive vs. negative effect of cited interactions.

**Condensing individual cognitive maps (ICMs)**

As respondents were allowed to describe the SES and its components in their own terms, a pre-processing *a posteriori* standardization of all cited components was necessary. To do so, we followed the following qualitative condensing procedure [8,9]:

- Synonyms and terms expressing the same idea were grouped, for example “stream” and “river” were grouped into the component named “Rivers”;
- Single terms expressing similar ideas were grouped, for example “protected area delineation” and “management of the protected area” were grouped into “Protected area creation”;
- When it did not alter the meaning of the respondents’ discourses, the least frequently cited components were merged with another more cited component;
- Terms used by only one respondent, terms not linked by any arrow in the cognitive map, or terms not related to an essential element regarding the research questions, were deleted when it did not significantly alter the meaning of the respondents’ discourses;
- When an interaction in a cognitive map expressed an essential idea that was depicted by a component in other maps, the interaction was changed into a component.

This procedure led to 162 unique components that were then grouped into 32 more general component types (see Table S2). This grouping was required in order to limit the complexity of the cognitive maps and ensure their interpretability and transparency [8,10,11]. To group the components into component types, we used the following rules:

- Component types must include important elements of Malagasy social-ecological systems (SES) in general, and of the Makay SES in particular, as known from previous studies. For example, “Species”, “Makay’s landscapes and habitats”, “Agriculture and husbandry” and “Geomorphological features” were part of these key elements;
- Components types corresponding to ‘Ecosystem services’ must follow the last version of the Common International Classification of Ecosystem Services (CICES; [12] to ensure a correspondence with an international standard and facilitate comparisons between studies;
- Components types belonging to the ‘Social components and processes’ category must reflect relevant stakeholders and social dynamics in the Makay SES, for example “Authorities”, “New stakeholders” and “Local people”;
- Component types must aggregate components that describe similar components and/or process. For example, “Law enforcement” includes “Local brigades” and “Security forces”;
- Component types must distinguish components and/or processes that are critical to the system and have contrasted interactions with other SES components. For example, “Fires” were too specific to be aggregated with other components, so they were included in a distinct component type.

We considered that the components cited by respondents, their standardization and regrouping into types should be context-specific in order to (i) not betray respondents’ discourses and opinions, (ii) take into account context-specific phenomena, and (iii) be adaptable to a variety of situations and SESs. One main strength of cognitive maps is to be able to deal with these context-specific considerations while allowing standardized and comparable analyses [8,13,14], so we argue it is important to not have a too constraining homogenization procedure. On the contrary, each component type was further associated with one of the 6 categories presented in S2 Table, which are relevant to virtually all SES and might facilitate comparisons between different SES.

S2 Table: Synthesis of all the words used by respondents during interviews (in French) and their regrouping into 162 unique components, 32 types of components and 6 categories.

| **Types of components** | **Components** | **Times cited** | **Terms used by respondents** |
| --- | --- | --- | --- |
| **Biophysical components and processes** | | | |
| Climate | Precipitation, floods and cyclones | 6 | crues ; crues et submersions ; crues violentes ; cyclones ; eaux torrentielles ; pluies torrentielles ; précipitations élevées |
|  | Droughts | 4 | assèchement ; baisse des précipitations ; sécheresses |
|  | Climate change | 3 | changement climatique |
|  | Climate in general | 2 | alternance des saisons ; chaleur et humidité |
|  | Dry season | 1 | saison sèche |
|  | Rain season | 1 | saison humide |
| Erosion processes | Soil erosion | 9 | érosion ; érosion des berges ; érosion des plateaux |
|  | Silting | 7 | ensablement ; ensablement et érosion |
|  | Landslides | 6 | éboulements ; éboulements, érosion ; perturbations naturelles, dynamique de la végétation, éboulis |
|  | Water run-off | 2 | ruissellement |
| Food production issues | Locust invasion | 2 | invasions de criquets |
|  | Water shortage | 1 | manque d'eau |
|  | Yield decrease | 1 | baisse des rendements |
| Geomorphological features | Canyons | 24 | canyons ; canyons (exposition différente) ; canyons (orientation, largeur) ; canyons et falaises ; canyons et rivières ; canyons étroits ou avec résurgences d'eau en hauteur ; canyons larges en aval de rivière ; canyons naturels ; canyons, montagnes ; géologie, canyons ; grandes falaises, canyons profonds ; petites vallées (canyons étroits) ; réseau de canyons ; rochers, canyons |
|  | Rivers | 20 | cours d'eau ; eau, rivières ; présence d'eau en fond de canyon ; rivières ; rivières (eau froide) ; rivières et nombreux points d'eau |
|  | Lakes | 12 | lac ; lacs ; lacs avec crocodiles ; lacs naturels autour de beronono ; retenues d'eau ; zones refuges profondes probables (lacs) |
|  | Detrital rocks | 11 | massif ; massif avec sable et couches d'argiles ; massif de grès non stable ; massif détritique ; massif détritique avec argiles ; massif géologique particulier détritique ; massif karstique ; massif rocheux grès ; roche gréseause; tas de sable, plateaux, labyrinthe, configuration spatiale |
|  | Caves | 10 | abris sous roche ; grottes ; grottes, canyons |
|  | Relief morphology | 9 | géologie : forme de cerveau ; géologie particulière ; géologie spéciale, paysage unique ; géologie, grès, relief spécifique ; labyrinthe de grès ; labyrinthe gorges enchevêtrées, cerveau ; massif inexploré, labyrinthe ; structure géologique ; topologie variée |
|  | Anthropogenic locks | 8 | blocs rocheux ; morphologie, enclavement, verrous ; obstacles ; parties de canyon fermées ; verrous anthropiques ; verrous anthropiques (physiques et culturels) ; verrous rocheux |
|  | Cliffs and uplands | 5 | crêtes (points de vue) ; falaises ; falaises et canyons ; falaises, relief, montagnes ; plateaux |
|  | Waterborne sediments | 5 | alluvions charriés ; argiles et calcaires ; matière organique, et sable ; sable |
|  | Water networks | 4 | beaucoup d'eau ; lacs et cours d'eau ; réseau d'eau ; sources d'eau |
|  | Watersheds | 4 | 3 bassins versants ; bassin versant ; bassin versant Est et nord-ouest |
|  | Substrate | 2 | sable ; substrat sable et roche |
|  | Isolated hills | 1 | collines isolées protégées du feu |
| Makay's landscapes and habitats | Forests | 21 | différents types de forets ; différents types de forets et écosystèmes ; Flore (forêts, canarium) ; foret ; forets ; forêts ; forets (dont menapanda) ; forêts avec micro-habitats ; forets de palmier ; forets humides, sèche, ripisylve ; forets intactes ; milieux fermés ; patchs de forets ; patchs de forets primaires ; petites forets ; petites forêts ; quelques forets |
|  | Makay’s landscapes and habitats | 9 | dynamiques des écosystèmes, mouvements entre canyons ; écosystèmes préservés et variés ; nature ; nature sauvage ; nature vierge ; paysages ; végétation ; vestiges de forets ; zone sauvage |
|  | Humid forest | 8 | essences de l'est de madagascar ; forets corridor ; forêts galerie sub-humides ; forets galeries ; forêts humides ; forêts humides (pandanus..) ; forêts humides galeries ; ilots de forets humides ; végétation berges : forets sub-humides ; zones humides: forets galerie |
|  | Dry forests | 7 | 3 fragments de forets sèches sur grands plateaux et forets sur collines isolées et autour du Makay ; foret de tapia ; foret sèche ; forêts denses sèches ; forets sèches ; forets xérophiles |
|  | Wetlands | 6 | écosystèmes humides ; zones humides ; zones humides, marécageuses |
|  | Dry vegetation | 4 | collines avec végétation sèche ; herbes sèches ; sommets arides avec plantes xérophyles ; végétation sèche dynamique saisonnière |
|  | Microhabitats | 4 | contrastes faune / flore, caractéritique de l'est et ouest du massif ; écosystèmes: différents habitats pour insectes et plantes ; gradient végétation ; gradients de végétation |
|  | Pioneer vegeration | 2 | espèces rares en bord de falaise ; végétation pionnière |
|  | Low-stratum vegeation | 1 | milieux ouverts |
| Species | Endemic and flagship species | 18 | animaux (lémuriens, oiseaux, rapaces, canards sauvages) ; biodiversité (faune / flore) : lémuriens, fosa, espèces menacées ; biodiversité : flore/faune (espèces menacées: lémuriens, fosa) ; biodiversité : lémuriens, oiseaux, espèces rares ; biodiversité animale riche (caméléons, serpents, grenouilles, oiseaux) ; biodiversité exceptionnelle, dont lémuriens et oiseaux ; biodiversité insectes, plantes, reptiles importante ; biodiversité spéciale ; biodiversité variée ; biodiversité végétale et animale, endémisme ; biodiversité végétale et animale, endémisme dont clé de voute ; biodiversité, endémisme, animaux ; écosystème, espèces en danger: tortue à grande tête... ; faune sauvage: oiseaux, lémuriens, crocodiles, sangliers, chauves souris, grenouilles ; grande diversité de plantes, endémisme ; lémuriens propithèques, caméléons furcifer ; lémuriens, oiseaux, rapaces ; oiseaux (les plus diversifiés) ; tortues |
|  | Aquatic species | 13 | anguilles, poissons ; biodiversité (dont espèces aquatiques: biodiversité faible) ; biodiversité aquatique ; faune piscicole et aquatique (pauvre en espèces endémiques) ; poissons ; poissons et insectes aquatiques ; poissons, lacs ; tilapias |
|  | Edible plants | 10 | espèces végétales domestiquées ; ignames ; palmistes ; plantes comestibles ; racines, fruits ; tubercules |
|  | Terrestrial common species | 10 | animaux ; animaux divers: insectes, autres mammifères, fosa, crocodiles... ; biodiversité, faune, flore ; carnivores ; cochons sauvages ; faune et flore malgache classique ; faune: carnivores ; gros animaux ; lémuriens, potamochères ; oiseaux, lémuriens, tanrek, potamochère ; petits animaux ; populations végétales et animales de foret humide ; potamochères (tabous) ; potamochères invasifs ; proies |
|  | Medicinal plants | 7 | espèces récoltées: plantes médicinales ; plantes médicinales |
|  | Timber and construction materials | 6 | bois ; bois de chauffage ; bois de construction, plantes médicinales |
|  | Lemurs | 5 | lémuriens |
|  | Wild bees | 5 | abeilles sauvages ; essaims d'abeilles ; miel ; miel sauvage |
|  | Canarium | 4 | camphrier ; canarium ; canarium avec odeurs de camphre |
|  | Biodiversity in general | 3 | espèces: + 1300 ; hotspot biodiversité ; vestiges faune et flore |
|  | Biodiversity sink | 3 | refuge pour certaines espèces ; refuge pour les animaux ; réservoir de biodiversité |
|  | Invasive species | 3 | espèces exotiques ; petits bosquets avec espèces invasives (dégradé) ; plantes invasives |
|  | Varroa | 1 | varroa |
| Surrounding landscapes | Savannahs | 10 | désert, zone sèche ; milieu aride ; plaines collinéennes déboisées ; région aride ; savane ; savane ; savane, steppes avec jujubiers ; savanes ; savanes autour du Makay ; savanes autour du Makay, espaces herbeux |
|  | Human settlements | 2 | villages ; villages : sakoazoato (entrée), tsivoko |
| **Social components and processes** | | | |
| Access difficulties | Access difficulties | 20 | difficulté d'accès ; inaccessibilité ; inaccessibilité, peu de routes d'accès ; isolement ; isolement : pistes difficiles ; isolement, accès difficile ; isolement, accessibilité ; isolement, découverte ; isolement, inaccessibilité ; isolement, inaccessible ; isolement, reculé ; localisation : difficile d'accès, éloignement ; pas de sentiers dans le Makay ; zone enclavée, difficile accès |
| Agriculture and husbandry | Zebu raising | 28 | bétail, zébus ; élevage de zébus ; élevage et vente des zébus ; élevage zébus ; peu de zébus dans le Makay ; troupeaux ; zébus |
|  | Pasturelands | 24 | espaces de pâturages ; herbes brulées ; pâturage dans l'AP ; pâturage des zébus ; pâturages ; pâturages ; pâturages zébus ; prairies ; terres de pâturage ; terres de pâturages ; zones de pâturage |
|  | Rice cultivation | 21 | cultures bords rivières ; quelques rizières, peu d'agriculture ; riziculture ; riziculture inefficace (1 récolte par an), agriculture non diversifiée ; rizières ; rizières irriguées ; rizières, agriculture irriguée |
|  | Other crop cultivation | 18 | Agriculture ; agriculture extensive sur brûlis ; agriculture non irriguée ; agriculture sur brulis ; agriculture sur brûlis ; arbres fruitiers ; autres cultures ; autres cultures: maraichage... ; culture de maïs ; culture manioc ; cultures ; cultures de manioc, patates douces ; maraichage ; cultures manioc ; cultures sur brulis ; cultures vivrières ; terres agricoles |
|  | Small-scale breeding | 4 | moutons, volailles ; pisciculture ; Porcs, poulets, canards ; poules ; poulets |
|  | Agriculture in general | 1 | élevage de zébus et agriculture |
| Authorities | Traditional authorities | 7 | autorités coutumières ; autorités traditionnelles ; chefs de région ; chefs traditionnels ; roi ; rois |
|  | Local authorities in general | 6 | autorités locales ; autorités locales : administratif / coutumier |
|  | Decentralized administration | 5 | autorités administratives ; autorités décentralisées ; Autorités locales et régionales ; collectivités décentralisées ; maire |
|  | Malagasy State | 3 | Etat ; Etat, armée |
|  | Ministry of mines | 3 | ministère des mines |
|  | National authorities | 3 | Etat ; Etat, armée |
|  | Ministry of culture and environment | 2 | ministère de la culture et du patrimoine ; ministère de l'environnement ; services régionaux (dreef), ministère de l'environnement (Meef) |
| Cultural features | Cave paintings and archeological remains | 17 | cupules (trous dans les rochers faits par l'homme) ; grottes ornées ; peintures et vestiges archéologiques ; peintures pariétales récentes (18-19è siècles) ; peintures rupestres ; poteries, graines oléagineuses consommables, ossements de zébus ; sites archéologiques |
|  | Graves | 16 | grottes sépultures ; sépultures ; sépultures abandonnées ; sépultures actuelles ; site funéraire: tombeaux ; sites culturels : tombeaux ; tombeaux ; tombes |
|  | Cultural features in general | 1 | tombes et peintures rupestres |
| Demographic processes | In-migrations | 7 | dynamiques externes d'occupation et de mise en valeur ; immigrants ; migrations entre villages ; nomades "chasseurs-cueilleurs" ; pêcheurs (viennent de loin) ; populations extérieures ; populations nomades |
|  | Increase in land pressure | 4 | augmentation des besoins en terres ; création de nouvelles terres de culture ; dynamiques internes de créations d'espaces habités et cultivés ; pression foncière |
|  | Population increase | 2 | accroissement population ; démographie : augmentation population |
|  | Increase in water demand | 1 | besoins en eau croissants |
|  | Out-migrations | 1 | migrations sortantes |
| Environmental projects funding | funding through tourism | 3 | financement ; paiement de droits d'entrée |
|  | funding through donations | 2 | donateurs ; projet plus action, hello asso |
| Fear of external stakeholders | Fear of external stakeholders | 3 | peur ; peur des inconnus |
| Food security issues | Lean season | 5 | insécurité alimentaire, périodes de soudure ; périodes de soudure |
|  | General food issues | 1 | problématiques d'alimentation |
| Income for local people | incomes for local people | 15 | apport de nouveaux revenus ; apport de revenus ; arrivée de nouveaux revenus ; augmentation des tarifs, taxes ; création d'emplois ; création d'emplois, revenus ; création emplois ; source d'activité ; source de revenus ; source finanière |
| Insecurity | General insecurity | 9 | insécurité ; insécurité : vol de zébus et enlèvements ; insécurité grandissante |
|  | Zebu theft | 6 | violence ; vol de zébu à grande échelle ; vol de zébus ; vol zébus (et autres ) ; vols ; vols de zébus |
|  | Banditry | 1 | banditisme |
| Law enforcement | Security forces | 6 | armée ; militaires ; polices, gendarmerie |
|  | Local brigades | 2 | organisation face aux vols ; zama |
| Local people | Local people in general | 31 | éleveurs ; éleveurs de zébus ; habitants ; hommes et femmes ; pêcheurs locaux ; populations Bara ; populations locales ; populations locales (bara) ; populations locales : éleveurs et agriculteurs ; populations locales Bara ; populations locales des villages ; populations locales qui ne vont pas dans le Makay ; populations locales qui vont dans le makay: éleveurs, récolte... ; populations locales sédentarisées ; populations qui entrent dans le Makay ; populations qui n'entrent pas dans le Makay ; quelques villageois avec contacts en ville ; villageois ; villageois, guides locaux et porteurs |
|  | Zebu thieves | 19 | bandits ; dahalo ; voleurs de zébu ; voleurs de zébus |
|  | Local guides and workers | 17 | chauffeurs, guides, location maison, porteurs, cuisiniers ; guides locaux ; habitants impliqués dans actions de dvp: guides, pisteurs, gardiens ; locaux : porteurs, guides ... ; pisteurs et guides ; pisteurs, guides ; pisteurs, porteurs cuisiniers ; pisteurs, porteurs, cuisiniers locaux ; porteurs ; porteurs (porteuses), guides, cuisiniers ; porteurs, cuisiniers ; porteurs, cuisiniers, pisteurs ; porteurs, guides ; porteurs, pisteurs |
|  | Healers and soothsayers | 6 | devin guérisseur ; devins guérisseurs ; devins guérisseurs ; guérisseurs, maitres de rituel |
|  | Village associations | 3 | association d'apiculteurs de Beroroha ; associations locales ; associations villageoises |
| Media | Media | 6 | cinéastes ; communication, équipes tournage, dessinateurs ; diffusion de films ; équipes cinéma ; médias ; médias, télé |
| Movements within Makay | Temporary camps | 4 | campements temporaires (petites maisons) ; création de campements nomades dans les forets ; déplacement de familles et installation de camps temporaires ; déplacements de groupes de personnes vers le Makay et établissements temporaires |
|  | Permanent settlements | 3 | entrée de populations dans le Makay ; inhabité ; pas d'entrée de population/ zébus dans le Makay |
| New stakeholders | Tourists and ecovolunteers | 31 | écovolontaire ; écovolontaires ; écovolontaires et touristes ; touristes ; touristes et écovolontaires ; touristes malgache et internationaux ; touristes qui entrent dans le massif ; touristes qui restent au début des canyons ; touristes, randonneur ; visiteurs naturalistes et intéressés par la culture locale |
|  | Tour operators | 21 | agences touristiques ; investisseurs ; tours opérateurs ; tours opérateurs actuels ; tours opérateurs internationaux ; tours opérateurs locaux ; tours opérateurs malgaches et internationaux ; tours opérateurs nationaux ; tours opérateurs potentiels |
|  | Researchers and students | 20 | archéologues ; chercheurs ; chercheurs botanistes, entomologistes... ; chercheurs et stagiaires malgaches et étrangers ; chercheurs, étudiants, profs ; naturalistes et écovolontaires ; scientifiques ; scientifiques et étudiants ; scientifiques et Evrard |
|  | NGO Naturevolution | 13 | naturevolution ; naturévolution ; naturévolution france et Madagascar |
|  | NGO Naturevolution Madagascar | 12 | gestionnaire aire protégée ; gestionnaire délégué de l'Etat ; naturévolution mada ; naturevolution madagascar ; naturévolution madagascar ; Naturévolution Madagascar : gestionnaire ; Naturévolution Madagascar et malgaches des villes ; ONG gestionnaire ; ONG malgaches |
|  | NGO Naturevolution France | 10 | naturevolution France ; Naturévolution France ; naturévolution france (evrard) ; Naturévolution France : bailleur et promoteur ; ONG étrangères |
|  | Tourism-related stakeholders | 10 | arrivée d'étrangers, développement du tourisme ; arrivée du tourisme ; arrivée tourisme ; augmentation du tourisme ; chauffeurs avec 4X4 ; développement du tourisme ; développement touristique |
|  | New stakeholders in general | 5 | arrivée de personnes extérieures ; arrivée étrangers : touristes, chercheurs ... ; arrivée touristes et naturevolution ; attention attirée sur le Makay ; augmentation de la pression sur le Makay |
|  | Bernard Forgeau | 3 | Bernard ; Bernard et acteurs associés |
|  | NGO Action Makay | 2 | action Makay |
| Social conflicts | Social conflicts in general | 4 | conflits ; conflits inter et intra villages ; protestation ; rivalités |
|  | Conflicts caused by new stakeholders | 3 | conflits ; création de tensions, inégalités ; rivalités, révoltes |
|  | Conflicts caused by public policies | 2 | mécontentement ; résistance passive |
|  | Conflicts caused by zebu thiefs | 2 | conflits ; tensions entre villages |
|  | Conflicts caused by immigrants | 1 | conflit |
|  | Conflicts caused by Naturevolution | 1 | colère (politique) ; désaccords avec Naturevolution |
|  | Conflicts caused by tourism | 1 | mécontentement |
| Sociopolitical processes | Poverty and lack of education | 5 | appauvrissement ; dégradation du système éducatif et scolaire ; manque de connaissances ; manque d'éducation ; pauvreté, manque d'éducation |
|  | Retreat of national support | 5 | abandon par l'Etat et isolement des populations locales ; isolement, oublié de l'Etat ; recul de l'Etat ; recul de l'Etat, abandon, exactions |
|  | Changes in social organization | 4 | changement de la société traditionnelle ; dérangement des populations ; déstabilisation de l'organisation sociale ; échanges, cérémonies ; perte de savoirs traditionnels |
|  | Socioeconomic and political changes | 2 | changements macroéconomiques et politiques malgaches ; politique nationale et locale |
|  | Law enforcement | 1 | application des lois et règlements |
| **Ecosystem disservices** | | | |
| Ecosystem disservices | Fear of the Makay | 8 | croyances ; fady culturels ; peur ; peur d'entrer dans le Makay |
|  | Raptor attacks | 1 | attaques de rapaces |
| **Ecosystem services** | | | |
| Cultural ecosystem services | Recreational services | 27 | attraction (tourisme) ; attrait touristique ; aventure et exploration ; aventure: isolement, reliefs ; écotourisme ; observation des animaux et culture (potentiel futur) ; service récréationnel ; service récréatif ; service récréatif, terrain de jeu ; service récréationnel ; terrain de jeu pour aventuriers ; tourisme ; tourisme : aventure, découverte ; tourisme d'exploration ; tourisme, service récréatif et esthétique ; valeur esthétique et récréationnelle |
|  | Cultural services in general | 22 | aspect culturel ; aspect culturel, légendes, tabous, enterrements ; aspect spirituel: enterrements, peintures rupestres ; lieu de culte ; notion sacrée, aspect culturel fort ; recueillement, valeur spirituelle ; service culturel ; service culturel historique et actuel ; service spirituel ; services culturels, spirituel ; spirituel ; valeur culturelle ; valeur culturelle actuelle ; valeur culturelle passée ; valeur spirituelle et tabous |
|  | Zebu hiding and sheltering | 19 | abri / cachette zébus ; abri pour les zébus ; abri pour les zébus redevenus sauvages ; cachette de zébus ; cachette des zébus ; cachette et pâturage de zébus ; cachette zébus ; refuge de zébus volés ; refuge et pâturage pour les zébus ; refuge pour les zébus ; service culturel : dissimulation zébus ; traversée de zébus ; traversée des zébus ; traversée des zébus et cachette ; traversées de zébu ; traversées de zébus |
|  | Scientific interactions | 14 | collecte de données ; découvertes et recherches scientifiques ; découvertes scientifiques ; études scientifiques ; expéditions scientifiques naturalistes ; recherche scientifique ; recherches archéologiques ; recherches scientifiques ; recherches scientifiques sciences sociales ; service scientifique |
|  | Aesthetic services | 13 | beauté de l'endroit ; beauté des lieux ; beauté du lieu ; beauté du paysage ; beauté du site, somptueux ; beaux paysages ; esthétique |
|  | Burials | 9 | cimetière actuel ; cimetière ancien ; enterrement : spirituel ; enterrement des morts ; enterrement des morts, dimension sacrée, interdits ; enterrement des morts ; enterrements ; valeur culturelle, enterrement des morts |
|  | Natural heritage | 7 | patrimoine culturel ; patrimoine culturel et biologique du pays ; patrimoine culturel et historique ; patrimoine naturel ; valeur culturelle ; valeur culturelle nationale ancestrale ; valeur d'existence |
|  | Shelters for humans | 2 | lieu de refuge, fuite (passé) ; refuge et cachette pour les hommes |
|  | Local livelihoods maintenance | 1 | cohésion sociale, mode de vie, éloignement du village |
| Provisioning ecosystem services | Water provisioning | 25 | apport d'eau ; apport eau ; approvisionnement eau ; approvisionnement eau : boisson, usage quotidien ; approvisionnement en eau ; eau de consommation: se laver, laver vêtements, boire ; eau d'irrigation ; fourniture en eau ; réservoir d'eau ; réservoir d'eau, château d'eau ; réservoir d'eau, éponge ; réservoir hydrique ; source d'eau |
|  | Hunting | 18 | approvisionnement nourriture lémuriens ; braconnage lémuriens ; chasse ; chasse des lémuriens, oiseaux, insectes ; chasse et pêche ; chasse potamochère, lémurien ; fourniture nourriture: chasse |
|  | Gathering | 15 | approvisionnement nourriture : tubercules ; approvisionnement ignames ; approvisionnement nourriture (cueillette) ; collecte igname ; cueillette ; cueillette de tubercules ; cueillette ignames ; récolte de patates sauvages ; récolte de tavolo ; récolte de tavolo et autres tubercules, palmier ; récolte de tubercules |
|  | Fishing | 14 | pêche ; pêche: poissons, crustacés ; pêcheurs |
|  | Timber extraction | 11 | exploitants bois ; exploitation humaine |
|  | Honey collection | 10 | apicueillette ; approvisionnement miel ; prélèvement de miel (apicueillette) ; prélèvements de miel ; récolte de miel |
|  | Medicinal plant collection | 6 | approvisionnement plantes ; approvisionnement plantes med ; collecte de plantes médicinales ; collecte plantes médicinales ; récolte de plantes médicinales |
|  | General food provisioning | 4 | approvisionnement nourriture : chasse,, pêche, ignames ; approvisionnement en nourriture ; cueillette et chasse ; un peu de chasse et cueillette |
|  | General provisioning | 4 | chasse potamochères, lémuriens, apicueillette, agrumes, pastèques, plantes med, canarium ; collectes de plantes médicinales, vannerie miel, écrevisses, anguilles ; cueillette ; cueillette ignames, bois, vannerie, miel |
|  | Tree resin harvest | 2 | récolte de résine (encens dans les églises) ; récolte de résine pour l'encens |
| Regulating ecosystem services | Erosion control | 9 | lutte contre l'érosion ; service de lutte contre l'érosion ; service de régulation de l'érosion |
|  | Seed dispersal and polination | 5 | dispersion graines ; dissémination et pollinisation ; pollinisation ; pollinisation / dispersion des graines |
|  | Water quality regulation | 5 | épuration de l'eau ; filtration de l'eau ; qualité de l'eau ; régulation qualité de l'eau |
|  | Climate regulation | 3 | fourniture d'O2 ; régulation climat ; régulation climat: puits carbone |
|  | Rainfall regulation | 3 | évapotranspiration, régulation du cycle de l'eau ; évapotranspiration, pluie plus fréquente ; régulation des précipitations |
|  | Sedimentation | 1 | apport sédiments |
|  | Water flows regulation | 1 | régulation du débit des rivières |
|  | Zebu fertility | 1 | fécondité des zébus (car régulation des maladies des zébus) |
| **Negative human interventions** | | | |
| Environmental degradation | Deforestation | 13 | coupes de bois ; déforestation ; défriche ; défrichement ; ouverture des forets et assèchement ; pas de régénération ; perte d'habitats ; problèmes écologiques, destruction des habitats |
|  | General environmental degradation | 5 | augmentation des impacts des populations locales sur le Makay, activités non durables ; dégradation de l'environnement ; dégradation de l'environnement à proximité des villages ; dégradation de l'environnement naturel ; impacts sur le biotope ; pression anthropique |
|  | Pollution | 5 | déchets ; déchets, nuisances sonores, dégradations ; perturbation des espèces et pollution (sonore, déchets, visuelle) ; pollution ; pollution de l'eau |
|  | Trampling and overgrazing | 3 | piétinement ; surpâturage |
|  | Loss of aquatic habitats | 2 | assèchement des eaux saisonnier ; assèchement en cours |
|  | Disease outbreak | 1 | apport de maladies |
| Fires | fires | 31 | feu ; feux ; feux de brousse ; progression feux de brousse |
| Resource external extraction | Mining resources | 5 | pétrole, uranium, saphir, bois ; ressources minières ; ressources minières : uranium et pétrole ; richesses minières |
|  | Mining | 4 | extracteurs miniers ; extraction artisanale d'or ; industriels ; sociétés minières ; trafic |
|  | Mining exploration | 3 | explorations minières ; prospections ; prospections minières |
|  | Wildlife illegal trade | 2 | capture de lémuriens pour animaux de compagnie ; trafiquants |
|  | Wood harvest | 2 | approvisionnement bois ; bois chauffage et construction ; bois construction (grands arbres) ; bois de chauffe ; collecte ; collecte bois ; collecte de bois (construction et cuisine) ; collecte de bois construction et chauffe ; coupe de bois ; coupe de bois construction, chauffage ; feuilles construction toitures, bois et bambous de construction maison et pour pilons |
|  | General external extractions | 1 | compagnies externes: minières, pétrolières, forestières |
|  | Mines | 1 | carrés miniers |
| **Positive human interventions** | | | |
| Conservation and development actions | Protected area creation | 15 | création aire protégée ; création AP ; création de l'aire protégée ; création de l'aire protégée, protection ; création de l'AP ; création de l'AP et protection ; délimitation de l'aire protégée ; dispositif de suivi et évaluation ; gestion de la zone ; mise en AP ; protection ; protection nature ; protection, gestion de l'AP |
|  | Environmental education and integration of local people | 13 | éducation ; éducation à l'environnement ; éducation des enfants et promotion d'autres pratiques agricoles ; formation ; intégration des populations dans la conservation ; sensibilisation ; sensibilisation à l'environnement ; sensibilisation, éducation à l'environnement des populations |
|  | Environmental projects around Makay | 5 | actions de conservation et développement ; conservation du Makay ; stratégie de gestion: mise en place de programmes de recherche, conservation, développement |
|  | beekeeping | 4 | projet d'apiculture ; projets apiculture |
|  | Reforestation | 4 | mise en place de pépinières ; projet de reforestation ; reboisement communautaire |
|  | Unspecified actions | 3 | mise en place de pépinières, apiculture, sensibilisation ; projets apiculture, reboisement, cultures alternatives, potager scolaire, grenier communautaire ; projets de conservation et développement ; projets de Naturevolution ; recherche d'alternatives moins impactantes pour l'environnement |

**From cognitive maps to adjacency matrices**

All standardized ICMs were exported from Mental Modeler as adjacency matrices. For a given ICM, its corresponding adjacency matrix was a square matrix of dimension equal to the number of components in the ICM. Each row and column was named after one component, and the value [*i,j*] in the matrix was either ‘0’ (no interaction cited by the respondent between component *i* and component *j*), ‘1’ (positive effect of component *i* on component *j*) or ‘-1’ (negative effect of component *i* on component *j*). Thus, adjacency matrices were not symmetric because the links between components were directed (*i* could influence *j*, but *j* did not necessarily influence *i*), and the diagonal was filled with zeros because a component cannot interact with itself.

Adjacency matrices were then augmented to allow their addition [8,9]. The non-cited components in a corresponding cognitive map were added in rows and columns filled with zeros.

**Social representations of the Makay SES**

*Background about social representations and their analysis*

To analyze the social representations of the SES, we relied on the structural approach of social representations [15,16]. This approach allows to explore the organization of the elements of the representations by distinguishing the central core elements of the representations from the peripheral elements. A commonly-used method for this approach is the free association, and in particular the multiple responses free association technique [4,5]. In this technique, the interviewer uses an inductive word or question, and the respondent is free to cite as many related items as desired, in general in a certain lapse time. This method is sometimes called free-listing and used to analyze cultural domains [17–19]. These techniques are considered to tap into the implicit meanings respondents attach to the stimulus, while being qualitative for respondents – who can express freely, without being constrained by pre-defined categories – and quantitative for researchers – who can perform statistical analyses [20].

To analyze the content and structure of social representations, two hierarchy indicators have been proposed by P. Vergès [21]: the frequency of a word and its rank of appearance during the free association exercise. The frequency gives access to a quantitative and collective dimension, thus constituting an indicator of quantitative centrality. The rank of appearance reflects the cognitive ability of the words, and relies on the assumption that that the words that appeared first are the most important for the respondent, which is sometimes a contested assumption. To overcome this criticism, Abric [15] proposed substituting this “appearance ranking” with an “importance ranking”: following the free association, the subject is asked to rank the words given according to their importance. Thus, this solution requires an extra step in the interview procedure, or ideally a second interview with each respondent that is not always possible due to a lack of availability of respondents or due to researchers’ own logistic constraints.

*The centrality-frequency method to study social representations*

We propose to use an alternative metric to assess the structure of social representations based on graph theory analysis. On top of the frequency of each cited item, we considered that centrality measures provided by graph theory analysis were a good estimate of the importance of cited items, as it reflects their centrality in the system described by respondents [8]. As a consequence, we developed a centrality-frequency method to analyze social representations, which is derived from the importance-frequency method. In graph theory analysis, the centrality of each node of a graph is the sum of all in-coming and out-coming edges [8]. We can also distinguish the outdegree (sum of all out-coming edges) from the indegree (sum of all in-coming edges), with the following relationship:

$Centrality = Outdegree+ Indegree.$

In other words, centrality gives information on nodes’ connectiveness and interacting power, reflecting their role and importance in the system and its dynamics, either as a receiver or as an influencer, or both [8,22]. Thus, it can be considered as an appropriate measure of the importance respondents assign to the different SES components they cited. However, centrality is also highly influenced by the total number of components cited by respondents (i.e. by the size of cognitive maps): the more a cognitive map contains nodes, the more one node can be linked to other nodes, and thus the higher centrality can be. To overcome this dependence between centrality and cognitive maps’ size, we used the centrality rank instead of the centrality itself to assess the importance people assigned to each map node. The rank of centrality has the advantage of being less dependent on map size, while still reflecting the place each respondent gave to each component in its representation of the SES.

In the centrality-frequency method, the elicitation procedure of people’s mental models of an SES is considered to be a free association technique without restriction that enables to identify all relevant items from inductive questions, followed by an assessment of the importance of each item based on their interactions with other items in the SES. The centrality-frequency method uses the occurrence frequency and the centrality score of each component as hierarchy indicators:

- The occurrence frequency (*f*) of a component *i* is the number of cognitive maps in which the component occurs (*N_i_*) related to the total number of cognitive maps (*N*): $f_{i}=\frac{N_{i}}{N}$. This frequency reflects the degree of consensus among respondents for this component;
- For each component *i*, in each ICM, we calculate the component’s centrality (sum of all in- and out-coming interactions) and rank all components according to their centrality; from all centrality rank thereby calculated for each ICM, we calculate the median centrality rank of the component *i*. As a result, components with a low median centrality rank are the components that generally occupy a central place in the ICMs, indicating that they are seen as key elements in the system by the respondents who cited them because they are perceived as having a high interactive power with other nodes in the ICMs. To avoid counter-intuitive formulations, we use the centrality score: a high centrality score reflects a low median centrality rank, and a low centrality score reflects a high median centrality rank.

*The different zones of social representations*

The combination of the two indicators allows distinguishing four sets of components in the social representations, as defined by Abric [15]:

- The **core zone** contains the most frequently cited and central components in the cognitive maps. This zone is believed to contain the elements that define and stabilize the social object, but also more secondary elements that have no significant values, such as synonyms or prototypical ideas associated with the social object. As Abric puts it, this zone contains the core of the social representation, but also less significant elements;
- The **contrasting elements** zone contains less frequently cited components, but central in the cognitive maps of those who have cited them. This zone can then reflect the existence of a minority that holds a different representation. In other words, this zone can depict the core zone of the representation held by a sub-group of respondents. Alternatively, this zone can also be seen as complementary to the first periphery, containing for examples details or generalizations, or highlighting the fact that respondents have different degrees of knowledge on certain components;
- The **first periphery** contains frequently cited elements situated in the periphery of the system. The components of this zone are likely to be consensual among respondents, but not central in the system;
- The **second periphery** contains less frequently cited and less central elements. They are peripheral both quantitatively and qualitatively in the social representations of the SES.

**Variability among individual representations**

To explore variability in respondent individual representations, we calculated for all respondents a set of 9 quantitative variables that depicted the structure and content of the ICMs (see S3 Table).

S3 Table: Variables used in the Principal Components Analysis (PCA)

| **Variables of the PCA** | **Description** | **Formula** |
| --- | --- | --- |
| **Active variables** |  |  |
| Number of components | Total number of components in the individual cognitive map (ICM) | N |
| Number of links | Total number of links in the ICM | C |
| Density | Ratio between the number of links in the ICM and all the possible links within a map containing the same number N of components | $\frac{C}{N(N-1)}$ |
| Categories of components  (6 variables in the PCA) | Proportion of components belonging to each of the six categories of components | $\frac{N_{\mathrm{cat}}}{N}$ |
| **Supplementary variable** |  |  |
| Respondent type | Predefined categories of respondents: researchers, project members, association members, tour operators |  |

**References**

1. Russell BH. Research Methods in Anthropology: qualitative and quantitative approaches. Fifth edit. Lanham, Maryland: AltaMira Press; 2011. 665 p.

2. Gray SA, Gray S, Cox LJ, Henly-Shepard S. Mental Modeler: A Fuzzy-Logic Cognitive Mapping Modeling Tool for Adaptive Environmental Management. In: 2013 46th Hawaii International Conference on System Sciences. IEEE; 2013. p. 965‑73.

3. Etienne M, du Toit DR, Pollard S. ARDI: A co-construction method for participatory modeling in natural resources management. Ecology and Society. 2011;16(1):44 [online].

4. Dany L, Urdapilleta I, Lo Monaco G. Free associations and social representations: some reflections on rank-frequency and importance-frequency methods. Quality & Quantity. 26 mars 2015;49(2):489‑507.

5. Lo Monaco G, Piermattéo A, Rateau P, Tavani JL. Methods for Studying the Structure of Social Representations: A Critical Review and Agenda for Future Research. Journal for the Theory of Social Behaviour. sept 2017;47(3):306‑31.

6. Costanza R, de Groot R, Braat L, Kubiszewski I, Fioramonti L, Sutton P, et al. Twenty years of ecosystem services: How far have we come and how far do we still need to go? Ecosystem Services. 2017;28:1‑16.

7. Rives F. Le concept de service écosystémique en écologie : émergence, utilisations, portée et controverses. Montpellier; 2013. (Serena). Report No.: n°2013-01.

8. Özesmi U, Özesmi SL. Ecological models based on people’s knowledge: a multi-step fuzzy cognitive mapping approach. Ecological Modelling. août 2004;176(1‑2):43‑64.

9. Mehryar S, Sliuzas R, Sharifi A, Reckien D, van Maarseveen M. A structured participatory method to support policy option analysis in a social-ecological system. Journal of Environmental Management. 2017;197:360‑72.

10. LaMere K, Mäntyniemi S, Vanhatalo J, Haapasaari P. Making the most of mental models: Advancing the methodology for mental model elicitation and documentation with expert stakeholders. Environmental Modelling & Software. févr 2020;124(November 2019):104589.

11. Elsawah S, Guillaume JHA, Filatova T, Rook J, Jakeman AJ. A methodology for eliciting, representing, and analysing stakeholder knowledge for decision making on complex socio-ecological systems: From cognitive maps to agent-based models. Journal of Environmental Management. mars 2015;151:500‑16.

12. Haines-Young R, Potschin MB. Common International Classification of Ecosystem Services (CICES) V5.1 and Guidance on the Application of the Revised Structure. 2018. p. 27.

13. Jones N a., Ross H, Lynam T, Perez P, Leitch A. Mental Models: An Interdisciplinary Synthesis of Theory and Methods. Ecology and Society. 2011;16(1):46 [online].

14. Moon K, Guerrero AM, Adams VanessaM, Biggs D, Blackman DA, Craven L, et al. Mental models for conservation research and practice. Conservation Letters. 7 mars 2019;(March 2018):e12642.

15. Abric J. La recherche du noyau central et de la zone muette des représentations sociales. In: Abric J-C, éditeur. Méthodes d’étude des représentations sociales. Erès « Hors collection »; 2005. p. 59‑80.

16. Abric J-C. L’approche structurale des représentations sociales: Développements récents. Psychologie & Société. 2001;4:81‑103.

17. Ryan GW, Nolan JM, Yoder PS. Successive Free Listing: Using Multiple Free Lists to Generate Explanatory Models. Fields Methods. 2000;12(2):83‑107.

18. Brewer DD. Supplementary Interviewing Techniques to Maximize Output in Free Listing Tasks. Fields Methods. 2002;14(1):108‑18.

19. de Albuquerque UP, de Lucena RF. Can apparency affect the use of plants by local people in tropical forests? Interciencia. 2005;30(8):506‑11.

20. Buijs AE, Elands BHM. Does expertise matter? An in-depth understanding of people’s structure of thoughts on nature and its management implications. Biological Conservation. déc 2013;168:184‑91.

21. Vergès P. Approche du noyau central: propriétés quantitatives et structurales. In: Guimelli C, éditeur. Structures et transformations des représentations sociales. Neuchâtel: Delachaux et Niestlé; 1994. p. 233‑53.

22. Moon K, Adams VM. Using quantitative influence diagrams to map natural resource managers’ mental models of invasive species management. Land Use Policy. janv 2016;50:341‑51.
