# Supplementary material for: Towards an inclusive nature conservation initiative: Preliminary assessment of stakeholders’ representations about the Makay region, Madagascar
Source: PLoS One. 2022 Aug 26;17(8):e0272223. doi: 10.1371/journal.pone.0272223 (PMC9417016; doi:10.1371/journal.pone.0272223)
Supplement: S2 Appendix — (DOCX) [file pone.0272223.s005.docx]

**S2 Appendix: Interview guide**

***Identification of the Makay’s characteristics and ecosystem services***

We are seeking for components in, and around, the Makay (natural living or non-living entities, natural or human-modified landscape features)

- What are the main characteristics of the Makay and its surroundings?
- What is in, and around, the Makay?

We are seeking for the benefits to any human group provided by the Makay region (living near the Makay or not, entering the Makay or not). These benefits can be material (e.g. resulting from climate or water cycle regulation) or non-material (e.g. aesthetic, spiritual, recreational, educational or scientific benefits).

- What are the ecosystem services provided by the Makay?
- What are the advantages or benefits provided by the studied area?
- What resources are collected in the Makay?

***Identification of the Makay stakeholders***

We are seeking for direct (those having a direct effect on the resources) and indirect stakeholders (those whose actions influence practices of direct stakeholders).

- Who are the people or groups of people playing a role in the dynamics of the territory or benefiting from the Makay?

***Identification of the dynamics, processes and drivers affecting the Makay***

We are seeking for ecological or social dynamics taking place in the Makay SES. These dynamics can be natural or anthropogenic; internal or external.

- What are the main dynamics affecting the Makay region (affecting stakeholders, components of the system…)?
- What are the drivers of change (or evolution) of the Makay region and its surroundings?

***Construction of links between the identified components***

We are trying to connect the components of the Makay SES, in order to show causal links between these components. These links can be positive or negative.

- What are the causal relationships between all the identified characteristics, ecosystem services, stakeholders and dynamics?

***Conclusion of the interview***

- Do you want to modify (add, remove or change) any components or links of the cognitive map you have built?
- Would you like to comment in more detail on the cognitive map?
