# Supplementary material for: Towards an inclusive nature conservation initiative: Preliminary assessment of stakeholders’ representations about the Makay region, Madagascar
Source: PLoS One. 2022 Aug 26;17(8):e0272223. doi: 10.1371/journal.pone.0272223 (PMC9417016; doi:10.1371/journal.pone.0272223)
Supplement: S1 Fig — (DOCX) [file pone.0272223.s006.docx]

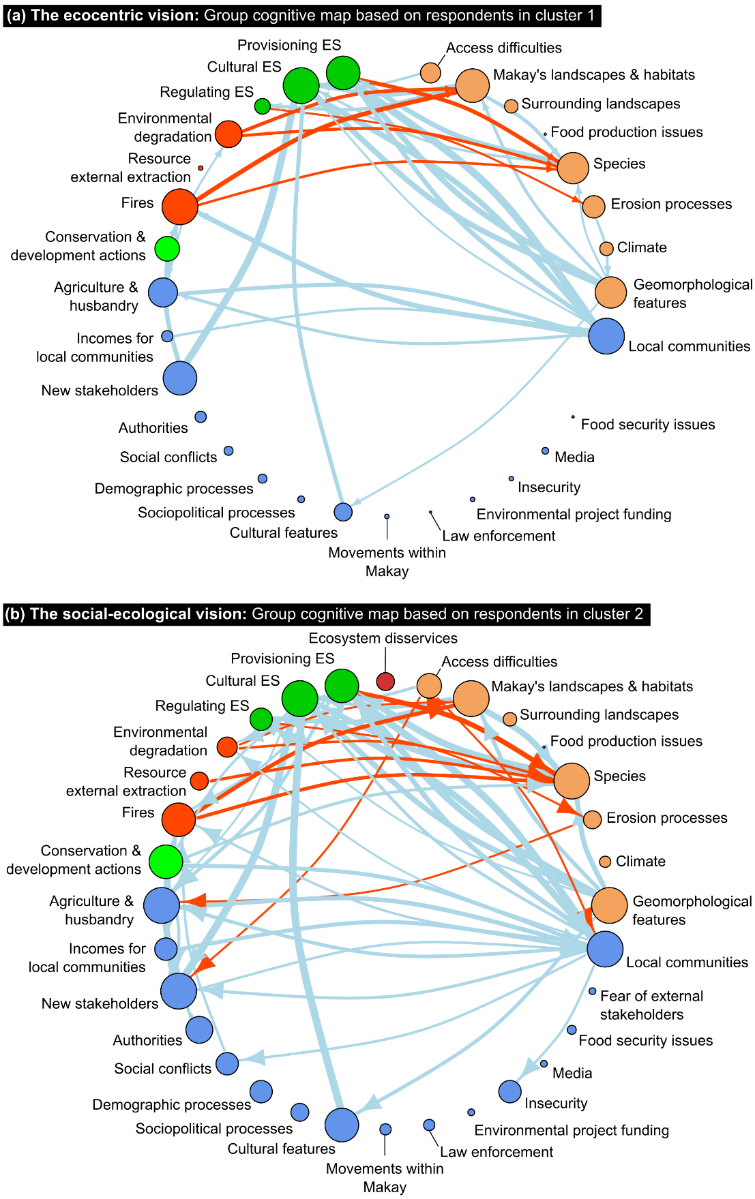


S3 Figure: Group cognitive maps of the Makay SES according to respondents’ distribution in the two clusters. (a) Map obtained from the individual cognitive maps of respondents in the ‘ecocentric’ cluster. (b) Map obtained from the individual cognitive maps of respondents in the ‘social-ecological’ cluster. Nodes correspond to the 32 component types, colored according to the category, with a size proportional to their frequency of citation in ICMs. Arrows correspond to the positive and negative interactions between component types, with a width proportional to their frequency of citation (for the sake of visibility, only interactions cited by >20% of respondents in each cluster are represented).
