# Supplementary material for: Towards an inclusive nature conservation initiative: Preliminary assessment of stakeholders’ representations about the Makay region, Madagascar
Source: PLoS One. 2022 Aug 26;17(8):e0272223. doi: 10.1371/journal.pone.0272223 (PMC9417016; doi:10.1371/journal.pone.0272223)
Supplement: S2 Fig — Graphs showing the elements of the four zones of the Makay social representations for (a) the ecocentric cluster, and (b) the social-ecological cluster. (DOCX) [file pone.0272223.s007.docx]

1. Social representation of the Makay of the ecocentric cluster  **(b)** Social representation of the Makay of the social-ecological cluster


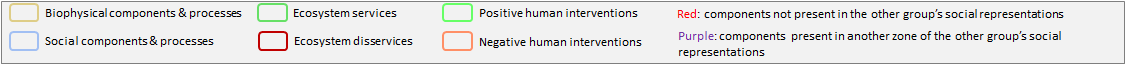

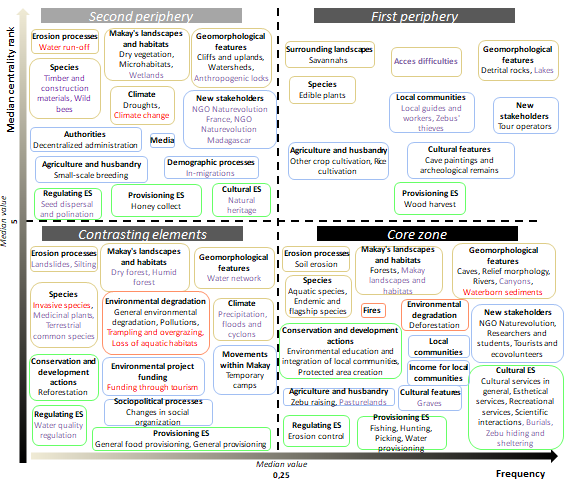

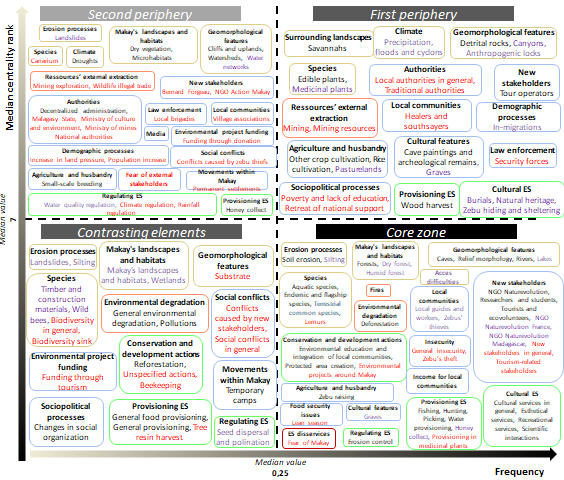


S4 Figure: Graphs showing the elements of the four zones of the Makay social representations for (a) the ecocentric cluster, and (b) the social-ecological cluster. The four zones were named following Abric (2005) and the classification of elements into the different zones was done with the centrality-frequency method. Boxes correspond to component types containing the components cited by respondents, and the color indicates the category. Only components cited by >10% of the respondents are represented.
