# Supplementary material for: Towards an inclusive nature conservation initiative: Preliminary assessment of stakeholders’ representations about the Makay region, Madagascar
Source: PLoS One. 2022 Aug 26;17(8):e0272223. doi: 10.1371/journal.pone.0272223 (PMC9417016; doi:10.1371/journal.pone.0272223)
Supplement: Attachment — Submitted filename: Response to reviewers.pdf [file pone.0272223.s008.pdf]

Response to Reviewers

Dear Dr. Rodrigo Pereira Medeiros,

First and foremost, we would like to thank you for giving us the opportunity to submit a revised draft of our manuscript entitled " Towards an inclusive nature conservation initiative: preliminary assessment of stakeholders' representations about the Makay region, Madagascar" to PLOS ONE. We appreciate the time and effort that you and the reviewers have dedicated to providing your insightful and constructive comments on our manuscript.

We have made changes to incorporate the suggestions provided by you and the reviewers. We have highlighted the changes within the manuscript in the attached document called "Revised Manuscript with Track Changes", and have detailed in a point-by-point our responses to the editor's and reviewers' comments and concerns. Finally, due to the substantial modification in the bibliography, we also revised the corresponding number of each reference cited in the paper.

We look forward to hearing from you regarding our submission and to responding to any further questions and comments you may have.

Sincerely,

Céline Fromont, April 28th 2022

Journal Requirements:

1. **PLOS ONE's style requirements**: We ensured that our manuscript meets PLOS ONE's style requirements.

2. **PLOS' questionnaire on inclusivity in global research**: We uploaded a completed version of our questionnaire as Supporting Information. Besides, we added the sentence "Additional information regarding the ethical, cultural, and scientific considerations specific to inclusivity in global research is included in the Supporting Information (S1 Checklist)" in a subsection 'Inclusivity in global research' to the Methods section.

3. **Acknowledgements and Funding Statement:** Thank you for your suggestion. As required, we removed the funding-related text from the acknowledgements, as follows:

"We thank the research laboratories SENS (UMR Knowledge, Environment and Societies), PALOC (UMR Local Heritage, Environment and Globalization), DIAL (UMR LEDa, DIAL) and Art-Dev (UMR Actors, Resources and Territories in Development) for their support. We would also like to thank all the stakeholders who accepted to be interviewed, and in particular Bernard Forgeau for its valuable help throughout this study."

We would like to update the Funding Statement as follows:

“SMC and CC received funding from the "Fondation Maison des Sciences de l'Homme" for the MakayMada project of which this study is part. The funders had no role in study design, data collection and analysis, decision to publish, or preparation of the manuscript”

4. **Data Availability statement:** Thank you for your suggestion, we do not wish to make changes to our Data Availability statement.

5. **Figure 1**: Thank you for being concerned by Figure 1. However, this figure does not use any copyrighted material. The map was built by Auréa Pottier (engineer at IRD), under QGIS, using OpenStreetMap data free to use under an open license. From these data, we made all the layers present on this map. We did not use any other sources for this map.

Accordingly, we made the changes in the caption as follows:

“Figure 1: Location of the Makay region, Madagascar. The map was built by Auréa Pottier (engineer at IRD), under QGIS, using OpenStreetMap data.”

6. **Figure 5**: Thank you for being concerned by Figure 5. However, this figure does not use any copyrighted material. It was built by the authors with R and Inkscape softwares, from our data. We did not use any other sources for this figure.

Comments from Reviewer #1:

Comment Rev1 #1: *I suggest to exclude the "presentation of the Malay region" as a new section clearly focused on the case study, and providing more details on the challenges and risks if the area is not protected.*

Response Rev1 #1: We agree with this comment and created a new section “Study site” (line 89) that provides more information about the Makay region. This section contains two subsections “Presentation of the Makay region” (line 90) and “Challenges and potential threats” (line 114) to give more information about the local and national context and potential risks if the area is not protected.

Comment Rev1 #2: *I suggest to organize the rest of the sub-divisions in just two main sub-sections: Data collection and Data analysis.*

Response Rev1 #2: We thank reviewer 1 for his suggestion. We have renamed the subsection “Elicitation of individual representations of the Makay” to “Data collection” (line 188), and merged the subsections “Condensing and aggregating individual cognitive maps” and “Analysis of social representations and ambiguity in individuals’ views” into “Data analysis” (line 240). Before these subsections, we have kept the "conceptual background" part, because it seems to us necessary as a preamble to explain the articulation of the mental-model theory and the social representation theory.

Comment Rev1 #3: *Furthermore, I think that sub-section 2.3 on Ethical approval should not be included in this section in the current detail, as the journal asked for ethical issues when submitting and as a separate issue.*

Response Rev1 #3: We followed PLOS ONE requirements and renamed this subsection "Inclusivity in global research" into the "Material and methods" section. We uploaded a completed version of our questionnaire on inclusivity in global research as Supporting Information (S1 Checklist).

Comment Rev1 #4: *Regarding data collection, I was wondering if 32 respondents are a validated sample to ensure that all confronted perspectives are consulted. For example, you specified their profile as researchers, members of NGOs (civil society) and tour operators... but what about managers? and public administration or government? They have no relevance in the decision-making process of the PA creation? The reasons to identify and select respondents must be clearly extended. Likewise, are interviews recorded? How about the transcription process? Does the speeches being codified or organized in some way to highlight shared views among respondents?*

Response Rev1 #4: We thank Reviewer 1 for pointing this out. In order to decide when to stop interviewing more respondents, we applied a data saturation technique that is commonly used in social investigations: we considered that enough respondents were interviewed when no new information (new components in cognitive maps) was added by an interview. We explain this data saturation in S1 Appendix (S1 Figure). Regarding the inclusion of managers, we included members of the NGO carrying the project of creation of the protected area and expected to be the future manager of this protected area among the members of local environmental organizations we interviewed. We have added this precision to line 199-200. Regarding public administration and government, we chose to elicit cognitive maps from people who had personally been in the Makay (we have added this precision to line 194), and public administration and government did not have sufficient on-the-ground knowledge of the region to produce a cognitive map of the Makay. However, we did meet with the Ministry of the Environment's Protected Area Creation and Management Department for additional interviews. The interviews were recorded but not transcribed in full, as our main material for data analysis was the cognitive maps produced by the respondents. We used an *a posteriori* standardization of all cited components in the cognitive maps to allow for analysis and comparisons between respondents. We used the recording of the interviews in order not to betray respondents' discourses and opinions. We give more information about this standardization procedure in the S1 Appendix.

Comment Rev1 #5: *In p. 18, line 245 it is presented a double focus on anthropogenic fires and provisioning ecoservices, but why both issues are considered to be focused in? The results section seems to identify both issues, so they are a result or a previous selection criteria to analyse data from the interviews?*

Response Rev1 #5: We thank Reviewer1 for raising this important point. We chose these two issues firstly because they were mentioned by most of the respondents and linked to many components of the Makay system, but also because these two issues are central in

Madagascar. We added some clarification in lines 414-416: “Besides, the problem of fires is well known in Madagascar [49–51], as is the importance of natural resources for the subsistence activities of local people [28].”, with the references “49. Kull CA. Madagascar aflame: landscape burning as peasant protest, resistance, or a resource management tool? Political Geography. 2002;21(7):927-53.”, “50. Frappier-Brinton T, Lehman SM. The burning island: Spatiotemporal patterns of fire occurrence in Madagascar. PLOS ONE [Internet]. 2022;17(3)” and “51. Kull CA. Deforestation, erosion, and fire: degradation myths in the environmental history of Madagascar. Environment and History. 2000;6(4):423-50.”

Comment Rev1 #6: *I just suggest to edit the title of sub-section 3.2 currently focus on ambiguity but to cognitive maps as the content is focused on that. Sub-section 3.3 seems a social-learning process so maybe the title "different perspectives" is so general...*

Response Rev1 #6: We changed the title of these subsections to “Variability in representations at individual and group level” and to “Complementary perspectives about Makay’s conservation challenges”.

Comment Rev1 #7: *In the discussion, sub-section 4.3 provides recommendations more than implications.*

Response Rev1 #7: We agree with Reviewer 1, and changed the title to “Recommendations for the Makay’s protected area”.

Comments from Reviewer #2:

We would like to thank Reviewer 2 for the time spent reviewing our manuscript and for his very positive comments on this work.
